# High risk for chikungunya virus to initiate an enzootic sylvatic cycle in the tropical Americas

**DOI:** 10.1371/journal.pntd.0005698

**Published:** 2017-06-29

**Authors:** Ricardo Lourenço-de-Oliveira, Anna-Bella Failloux

**Affiliations:** 1 Laboratório de Mosquitos Transmissores de Hematozoários, Instituto Oswaldo Cruz, Rio de Janeiro, Brazil; 2 Institut Pasteur, Arboviruses and Insect Vectors, Paris, France; University of Texas Medical Branch, UNITED STATES

## Abstract

**Background:**

Chikungunya virus (CHIKV) has dispersed in the Americas since 2013, and its range of distribution has overlapped large forested areas. Herein, we assess vector competence of two sylvatic Neotropical mosquito species, *Haemagogus leucocelaenus* and *Aedes terrens*, to evaluate the risk of CHIKV to initiate a sylvatic cycle in the continent.

**Methodology/Principal findings:**

*Haemagogus leucocelaenus* and *Ae*. *terrens* from the state of Rio de Janeiro, Brazil were orally challenged with the two CHIKV lineages circulating in the Americas. Fully engorged females were kept in incubators at 28±1°C and 70±10% humidity and examined at 3 and 7 days after virus exposure. Body (thorax plus abdomen), head and saliva samples were analyzed for respectively determining infection, dissemination and transmission. Both *Hg*. *leucocelaenus* and *Ae*. *terrens* exhibited high infection and dissemination rates with both CHIKV isolates at 7 dpi, demonstrating that they are susceptible to CHIKV, regardless of the lineage. Remarkably, *Hg*. *leucocelaenus* expectorated infectious viral particles as rapidly as 3 days after the infectious blood meal, displaying higher values of transmission rate and efficiency than *Ae*. *terrens*. Nevertheless, both species were competent to experimentally transmit both CHIKV genotypes, exhibiting vector competence similar to several American *Aedes aegypti*.

**Conclusions/Significance:**

These results point out the high risk for CHIKV to establish a sylvatic transmission cycle in the Americas, which could be a serious health issue as CHIKV would become another zoonotic infection difficult to control in the continent.

## Author summary

Chikungunya is a mosquito-borne-viral disease of African origin that has spread in the Americas since its first detection in 2013. The vector of Chikungunya virus (CHIKV) in the Americas is the mosquito *Aedes aegypti*. Due to this vector domestic behavior, CHIKV transmission is limited between this mosquito species and humans in urban and suburban American areas. However, since 2015 the distribution of CHIKV has moved towards the inland and the forested areas in the tropical Americas. The recent reports of CHIKV epidemic waves in zones intimately linked to the wild exemplify the potential of CHIKV initiating a zoonotic cycle in the continent since local sylvatic mosquitoes can be infected and transmit the virus. We experimentally demonstrated that two widely distributed American sylvatic primatophilic mosquito species, *Haemagogus leucocelaenus* and *Aedes terrens*, are highly susceptible and competent to transmit the two CHIKV lineages currently circulating in the continent, 7 days after an infectious blood meal, *Hg*. *leucocelaenus* mosquitoes presenting infectious viral particles in their saliva as rapidly as 3 days exposure. We concluded that there is a definite risk for CHIKV to establish a sylvatic cycle in the tropical Americas if local non-human primates can amplify the virus to infect wild primatophilic mosquitoes.

## Introduction

Several arboviruses of public health importance such as yellow fever virus (YFV), Chikungunya virus (CHIKV) and, more recently, Zika virus have spread from Africa to other continents. Coincidently in their historical cradle in Africa, these arboviruses are transmitted between non-human primates (NHP) by *Aedes* mosquitoes, mostly belonging to the Old World *Stegomyia* and *Diceromyia* subgenera [1]. Due to the spread of the anthropophilic mosquito *Ae*. (*Stg*.) *aegypti* outside Africa and the transit of viremic people, likely perpetrated by the globalization of trades and travel, these viruses have invaded several continents and are considered a most important alarming public health threat [2]. Owing to the domestic, synanthropic and anthropophilic behavior of *Ae*. *aegypti*, these arboviruses exploit urban and periurban ecosystems limiting transmission between *Ae*. *aegypti* and humans, originally in Africa and then the secondarily invaded continents such as Asia and the Americas [1, 2]. To date, the only documented exception is YFV (Flaviviridae: *Flavivirus*), which after migrating from Africa to the Americas, has spread into the forest in the tropical and subtropical areas. There, the YFV found susceptible NHPs capable of developing high viremia to infect Neotropical wild canopy-dwelling mosquitoes of the *Haemagogus* and *Sabethes* genera, initiating a sylvatic cycle [3]. Thereafter, YFV became a zoonotic arbovirus in the Americas as it was originally in Africa. The urban transmission of YFV has been eradicated from the Americas since the first half of the 20^th^ century, but each year YFV has victimized dozens of people in South America due to infections acquired from the sylvatic cycle. As YF vaccine is not adequately supplied and vaccination coverage is insufficient, the risk of YF transmission by the bite of infected wild mosquitoes remains a constant threat [3]. Moreover, as the control of the YFV enzootic circulation would be highly challenging, the enzootic spillover to an urban cycle has more than ever been feared due to great infestation by competent mosquito vectors—*Ae*. *aegypti* and *Ae*. *albopictus*—colonizing habitats near sylvatic foci [4,5,6].

Chikungunya virus (Alphavirus, Togaviridae) was first isolated in Tanzania in 1952 [7]. Three genotypes sharing a common ancestor in tropical Africa have been described: West African, East Central South African (ECSA) and Asian[8,9,10]. Like the YFV, CHIKV emerged from an enzootic cycle maintained between NHPs and sylvatic African mosquitoes, namely *Aedes furcifer*, *Aedes taylori*, *Aedes africanus*, *Aedes luteocephalus and Aedes neoafricanus* [11], establishing rural and urban cycles where *Ae*. *aegypti* ensured the inter-human transmission. Viremic people contributed to expand CHIKV territory to Asia, causing epidemics during the 1960’s and, since 2004–2005, a pandemic covering the Indian Ocean region, Asia, Mediterranean Europe and Central Africa [10,12]. These outbreaks were effected by the emerging Indian Ocean lineage (IOL), a monophyletic lineage descendant from the ECSA phylogroup, which contains a mutation in the envelope protein *(E1-Ala226Val)* that enhances viral transmission by the mosquito *Ae*. *albopictus* [9,10,13,14].

In the Americas however, autochthonous CHIKV transmission was only described in late 2013 in the Caribbean, the starting point of a large epidemic in the Americas [15, 16, 17]. From December 2013 to August 2014, nearly 660,000 cases were reported in the New World, and autochthonous transmission was confirmed to occur in 33 American countries and territories, 27 of which were in the Caribbean, while only French Guiana and Brazil reported CHIKV transmission in continental South America [15]. If in 2014 the CHIKV epidemic was primarily in the Caribbean, in 2015 the virus was identified in multiple countries of Central and South Americas with 30 countries or territories reporting CHIKV cases. In 2015, Colombia alone recorded 51.3% of the 693,000 cases in the Americas [18]. In 2016, South America reported 89.2% of the CHIKV cases in the Americas where the virus was detected in 42 countries or territories, including Brazil with nearly 76% of the 347,647 suspected cases recorded in the Americas [19]. Remarkably in 2015 and 2016, the distribution of CHIKV cases has progressively moved towards the inland and the forested areas resulting in a significant overlap with the area of the sylvatic YFV transmission cycle [20]. Thus, it is plausible that CHIKV viremic people infected in the *Ae*. *aegypti* urban cycle were bitten by sylvatic primatophilic mosquitoes in the neighboring forests or forest fringe. Since these mosquitoes are competent species transmitting a viral strain capable of being amplified by American NHPs or other vertebrates, CHIKV may initiate a sylvatic cycle as did the YFV in the past. Actually, the tropical American forest is presently more receptive to CHIKV transmission than ever due to the expanding and frequent CHIKV epidemic waves recently reported in zones contiguous to the wild.

Therefore, to assess the potential risk for CHIKV to establish a sylvatic transmission cycle in the Americas, we experimentally evaluated vector competence of two sylvatic primatophilic mosquito species, *Haemagogus leucocelaenus* (Dyar & Shannon) and *Aedes terrens* (Walker) for two CHIKV isolates belonging to the lineages currently circulating, ECSA and Asian genotypes [17].

## Materials and methods

### Mosquitoes

Female mosquitoes used in this study derived directly from field-collected eggs as *Hg*. *leucocelaenus* and *Ae*. *terrens* have never been successfully colonized in laboratory [21,22]. Eggs were collected in Parque Natural Municipal de Nova Iguaçu (PNMNI), an Atlantic forest conservation area in the State of Rio de Janeiro, Brazil (22°46’45”S 43°27’23”W), with 20 ovitraps suspended in the forest canopy at a height of 5-16m (median = 10m). Each ovitrap had three wooden paddles that were fortnightly changed from June to November 2016. The paddles were allowed to dry at room temperature, examined for egg number and stored in an insectary (26±1°C; 70±10% RH) until use. Eggs were hatched by immersing the paddles in dechlorinated tap water for two consecutive days. Larvae were reared in pans (~50 larvae/pan measuring 25x25x10cm) containing 1 liter of dechlorinated tap water, supplemented with yeast powder and shed leaves, renewed every 2–3 days. Adults were morphologically identified [23], kept in 30×30×30-cm mesh cages maintained in an insectary (28±1°C; 80±10% RH; 14h:10h light:dark cycle) and supplied with both 10% sucrose and honey solutions.

### Viral strains

Female mosquitoes were challenged with two CHIKV isolates belonging to two distinct lineages: CHIKV 05.115 (CHIKV_115) isolated from La Réunion in 2005 belonging to the ECSA lineage [24], and CHIKV_20235 (CHIKV_SM) isolated from Saint-Martin Island in 2013 and belonging to the Asian lineage [16]. They are phylogenetically related to strains circulating in Brazil and other American countries (17,25–28). CHIKV_115 and CHIKV_SM were isolated from human serum on *Ae*. *albopictus* C6/36 and Vero cells respectively, and viral stocks were produced following three passages in the respective cell lineage, then harvested and stored at -80°C until used for the mosquito experimental infection assays [16, 24]. Virus isolates were provided by the French National Reference Centers for Arbovirus at the Institut Pasteur in Paris and in Marseille.

### Mosquito experimental infection assays

Batches of 60 6–8 day-old female mosquitoes were isolated in feeding boxes and starved for 24 h, then exposed to the infectious blood-meal containing final viral titers of 10^7.5^ PFU/mL (CHIKV_115) and 10^6.5^ PFU/mL (CHIKV_SM), which correspond exactly to the same titers, passage and stocks used by Vega-Rua et al. [29,30] to assess vector competence of American *Ae*. *aegypti* and *Ae*. *albopictus* populations. The infectious meal consisted of a mixture of two parts of washed rabbit erythrocytes and one part of the viral suspension. Females were fed through a pig-gut membrane and the infectious blood-meal was maintained at 37°C. Mosquito feeding was limited to 1 hour. Only fully engorged females were incubated at 28°C constant temperature, 80% RH and 14h:10h light:dark cycle, with unlimited access to 10% sucrose solution [31]. As expected for sylvatic, not colonized mosquitoes, the artificial blood-feeding rates under experimental conditions here were low (<10%). Thus when available, samples of around 20 mosquitoes of each species were examined at 3 and 7 days after virus exposure, abbreviated as “dpi”. Females were individually processed as follows: abdomen and thorax (herein after referred to as body) were examined to estimate viral infection rate, head for viral dissemination and saliva for viral transmission [31]. For the determination of viral infection and viral dissemination rate, each mosquito body and head were respectively ground in 500 μL and 250μL of Leibovitz L15 medium (Invitrogen) supplemented with 2% fetal bovine serum (FBS; Eurobio) and centrifuged at 10,000 x *g* for 5min at +4°C for further inoculation onto monolayers of *Ae*. *albopictus* C6/36 cell (Institut Pasteur, Paris) culture in 96-well plates [29,31]. After 1 h incubation of homogenates at 28°C, 150 μL of 2.4% CMC (carboxymethyl cellulose) in Leibovitz L15 medium supplemented with 10% FBS was added per well. After 3 days of incubation at 28°C, cells were fixed with 10% formaldehyde, washed and revealed with hyperimmune ascetic fluid specific to CHIKV as the primary antibody and Alexa Fluor 488 goat anti-mouse IgG as the second antibody (Life Technologies). Presence of viral particles was assessed by detection of focus forming units (FFU). To estimate viral transmission, mosquito saliva was collected in individual pipette tips containing 5 μL FBS for 30 min as previously described [32]. Then, FBS containing mosquito saliva was expelled into 45 μL of Leibovitz L15 medium for titration in *Ae*. *albopictus* C6/36 cell culture in 96-well plates and stained as described above. Viral load in saliva was expressed as FFU/saliva.

Infection rate (IR) refers to the proportion of mosquitoes with infected body among tested females. Disseminated infection rate (DIR) corresponds to the proportion of mosquitoes with infected head among the previously detected infected mosquitoes (i.e, abdomen/thorax positive). Transmission rate (TR) represents the proportion of mosquitoes with infectious saliva among mosquitoes with disseminated infection. Transmission efficiency (TE) represents the proportion of mosquitoes with infectious saliva among the initial number of females tested [29].

### Statistical analysis

We used the Wilcoxon signed rank test to compare the viral load in the saliva. Significant difference was established when p-values were lower than 0.05. Data analyses and graphics were done with PRISM 5.0 software (GraphPad Software, San Diego-CA, USA, 2007).

### Ethics statements

The Institut Pasteur animal facility has been accredited by the French Ministry of Agriculture to perform experiments on live animals in compliance with the French and European regulations on care and protection of laboratory animals (directive 2010/63/EU). This study was approved by the Institutional Animal Care and Use Committee (IACUC) at the Institut Pasteur and by the Institutional Ethics Committee on Animal Use (CEUA-IOC license LW-34/14) at the Instituto Oswaldo Cruz. Mosquito collections in the Atlantic forest were approved by local environmental authorities (PNMNI license 001/14-15; SISBIO-MMA licenses 37362–2 and 012/2016). This study did not involve endangered or protected species.

## Results

**Fig 1:**
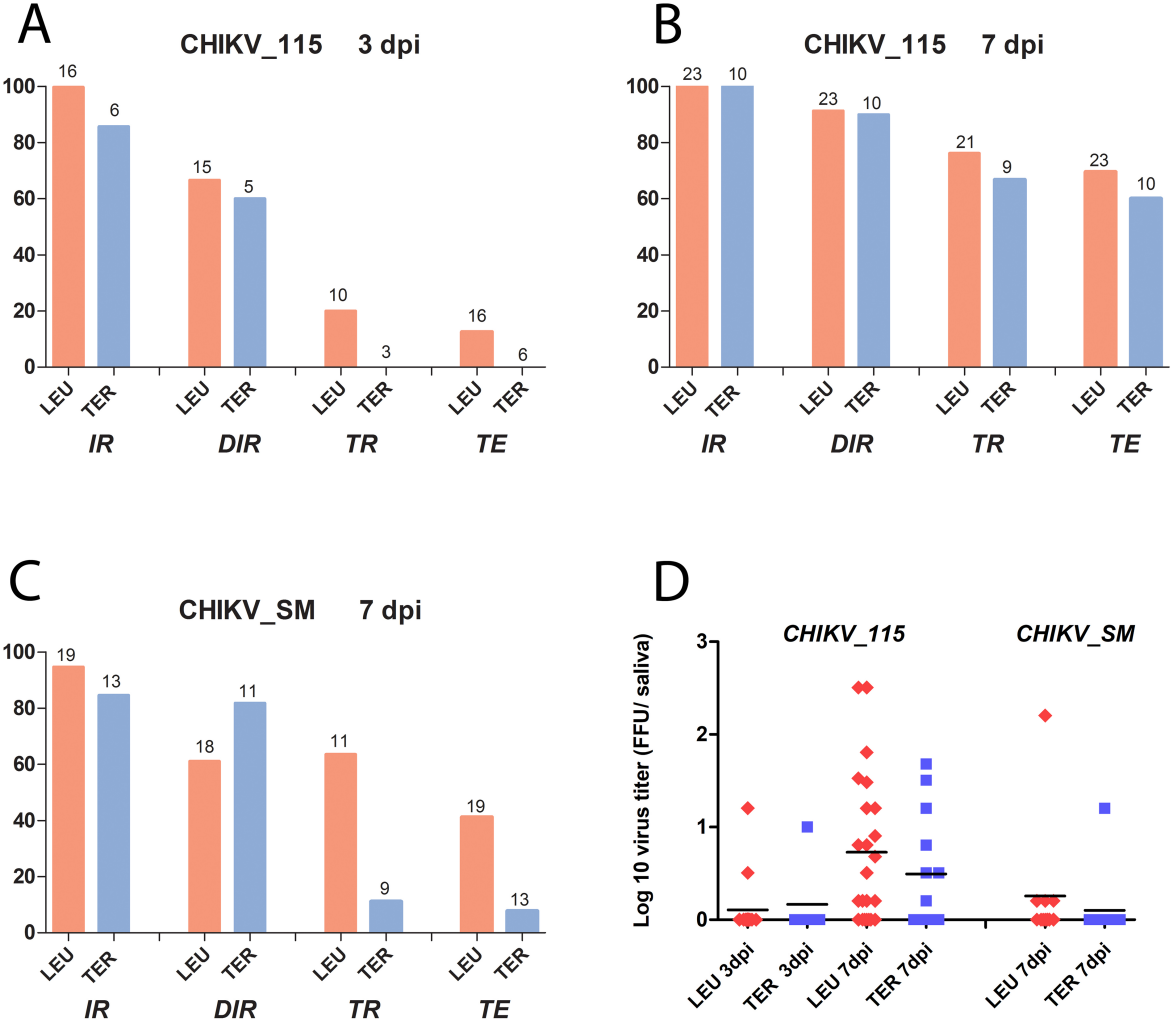
Viral infection, dissemination, transmission (A-C) and saliva viral loads (D) at 3 and 7 days after *Haemagogus leucocelaenus* (LEU) and *Aedes terrens* (TER) from Rio de Janeiro, Brazil, challenge with two CHIKV isolates of the two genotypes currently circulating in the Americas, CHIKV-115 (ESCA genotype) and CHIKV_SM (Asian genotype) when provided at a titer of 10^7.5^ PFU/mL and 10^6.5^ PFU/mL, respectively. Infection rate (IR) refers to the proportion of mosquitoes with infected bodies among tested females. Disseminated infection rate (DIR) corresponds to the proportion of mosquitoes with infected heads among the previously detected infected mosquitoes (i.e, abdomen/thorax positive). Transmission rate (TR) represents the proportion of mosquitoes with infectious saliva among mosquitoes with disseminated infection. Transmission efficiency (TE) represents the proportion of mosquitoes with infectious saliva among the initial number of females tested [29]. The number of mosquitoes analyzed for each vector competence rate is given on top of bars. The horizontal black bars (in panel D) represent the mean viral loads.

Both *Hg*. *leucocelaenus* and *Ae*. *terrens* exhibited high infection and dissemination rates with both CHIKV isolates at 7 dpi, demonstrating that they are CHIKV susceptible, regardless of the lineage (Fig 1A–1C). Indeed, 99.7% *Hg*. *leucocelaenus* and 85.7% *Ae*. *terrens* were already infected with the CHIKV_115 at 3 dpi, when 66.6 and 60% had disseminated infection, respectively. As expected, viral dissemination increased from 3 dpi to 7dpi (Fig 1A and 1B), exceeding 90% in both species when infected with the ESCA isolate (CHIK_115). Even with the Asian genotype (CHIK_SM) delivered at a lower dose, the two mosquito species also presented high IR (94.7% in *Hg*. *leucocelaenus* and 84.6% in *Ae*. *terrens*) and DIR (61.1% and 81.8%, respectively) (Fig 1C).

Most importantly, both *Hg*. *leucocelaenus* and *Ae*. *terrens* were competent to transmit CHIKV of both lineages circulating in the Americas at 7 dpi. Moreover, *Hg*. *leucocelaenus* was able to transmit infectious viral particles as rapidly as 3 dpi (Fig 1A). It usually exhibited higher TE and TR than *Ae*. *terrens* regardless of the CHIKV lineage and time of incubation (Fig 1A–1C). Nonetheless, one week after ingesting the infectious meal with the CHIKV_155, TE and TR varied from 60 to 66.6% in *Ae*. *terrens* and reached values as high as 69.5 to 76.2% in *Hg*. *leucocelaenus*. When considering the CHIKV_SM delivered at a lower dose to mosquitoes, TR was still high (63.6%) in *Hg*. *leucocelaenus* while it dropped to 11.1% in *Ae*. *terrens* at 7 dpi (Fig 1B and 1C). At 7 dpi, the saliva viral load ranged from 0.2 to 2.5 log_10_ for *H*g. *leucocelaenus* and from 0.2 to 1.6 log_10_ for *Ae*. *terrens* when infected with the CHIKV_115, and from 0.2 to 2.2 log_10_ for *H*g. *leucocelaenus* when infected with the CHIKV_SM. When infected with the CHIKV_115, the saliva viral load(Fig 1D) did not differ between species regardless of the incubation period (p = 1.00 and p = 0.151, for 3 and 7 dpi, respectively). However, the saliva viral load was higher in *H*g. *leucocelaenus* than *Ae*. *terrens* when infected with CHIKV_SM (p = 0.01). When evaluating the same mosquito species, the saliva viral load did not differ between the two CHIKV isolates, but when infected with the CHIKV_115, the expectorated saliva viral load was higher in *H*g. *leucocelaenus* at 7 dpi than at 3 dpi (p = 0.008).

## Discussion

We demonstrated for the first time that two sylvatic primatophilic mosquito species from the Americas are competent to transmit CHIKV belonging to the two lineages in circulation. High TRs were detected for both *H*g. *leucocelaenus* and *Ae*. *terrens* one week after ingesting infectious blood meals containing viral titters close to viremia of CHIKV-infected patients [33]. Importantly, infectious viral particles were detected as rapidly as 3 dpi in the saliva of *H*g. *leucocelaenus* challenged with the ESCA isolate (CHIK_115) which is the major lineage circulating in the current Brazilian epidemics, including the area of Rio de Janeiro where the tested mosquito population was originated [17,25,26,27,28]. Moreover, values of vector competence estimated herein for both *H*g. *leucocelaenus* and *Ae*. *terrens* were similar to those described for *Ae*. *aegypti* and *Ae*. *albopictus* populations from tropical Americas and the Caribbean, challenged with the same viral strains and titers by Vega-Rua et al.[29,30]. Such a comparison however, should be considered with caution due to the low number of mosquitoes examined, especially for the *Ae*. *terrens*.

Both *H*g. *leucocelaenus* and *Ae*. *terrens* are arboreal, tree hole breeding mosquitoes that can bite not only NHPs in the forest canopy, but also humans at the ground level [34–42]. Besides, both mosquitoes are capable of flying long distances linking isolated patches of the forest and bite people in the open fields, particularly in the ecotone between the wild and the man-modified environment [23,43,44]. For instance, *H*g. *leucocelaenus* frequently bite humans both in primary and secondary growing forests as well as in the peridomicile located far from forests [45,46]. All together, these patterns of behavior favor the zoonotic transmission of arboviruses by these mosquitoes to humans and vice-versa. Indeed, *H*g. *leucocelaenus* has been proven to play an important role in the transmission of sylvatic YFV as well as other human-infecting arboviruses across the Americas [36,39,47,48]. This mosquito has been incriminated as the primary vector in the recent YFV epizooties and epidemics reported in Southern and Southeastern Brazil [49,50]. Notably, both *H*g. *leucocelaenus* and *Ae*. *terrens* have a large geographical distribution in the New World. *Ae*. *terrens* has been reported from Mexico to northern Argentina (Argentina, Bolivia, Brazil, Colombia, Costa Rica, Ecuador, French Guiana, Guatemala, Guyana, Mexico, Panama, Paraguay, Suriname and Venezuela), and *H*g. *leucocelaenus* from Panama to northern Argentina and Uruguay (Argentina, Bolivia, Brazil, Colombia, French Guiana, Guyana, Panama, Paraguay, Peru, Suriname, Trinidad and Tobago, Uruguay and Venezuela) [35,36,51].

The ability to experimentally transmit the two lineages of CHIKV circulating in the Americas described herein for *H*g. *leucocelaenus* and *Ae*. *terrens* might become a pivotal factor facilitating the establishment of CHIKV in a zoonotic cycle in the Americas. The current CHIKV outbreaks in the continental tropical Americas covering a large geographical range of competent sylvatic mosquitoes enhance the chances of CHIKV to approach the forests. Indeed, the transit of CHIKV viremic people combined with the vector competence of *H*g. *leucocelaenus* and *Ae*. *terrens*, the large distribution of vectors and their biological and behavioural features suggests potential for CHIKV to become zoonotic in the continent. Additionally, high densities of *Ae*. *albopictus* populations, experimentally CHIKV transmission competent, in the transition zone between urban and forest environments possibly favor this phenomenon as well [29,52].

However, the establishment of a CHIKV sylvatic transmission cycle in the Americas depends upon the susceptibility of local NHPs to this virus, which has not been assessed yet. Wild NHPs were detected naturally infected with CHIKV in Malaysia, which suggested that a sylvatic, zoonotic transmission cycle could also occur in Asia [53]. Therefore, if Neotropical NHPs can amplify CHIKV and produce sufficient viremia to infect mosquitoes, the establishment of a sylvatic CHIKV cycle in the Americas could occur. The establishment of a CHIKV sylvatic transmission cycle in the New World would have immediate public health consequences as, so far, there are no efficient methods to control the enzootic circulation of any arboviruses. As learned from the case of YFV, the control of a potential zoonotic transmission of CHIKV in the Americas would be extremely challenging. An effective fight against tree hole harboring, canopy feeding mosquito vectors is not feasible, and there is still neither antiviral treatment nor a licensed vaccine to prevent infection in the case of CHIKV [54]. Thus, surveillance programs need to be organized in the continent to determine whether CHIKV has initiated a sustainable zoonotic transmission, which should search for natural infections in NHPs and enzootic vectors, together with the investigation of neutralizing antibodies in NHPs and other sentinel vertebrates as well as people living near forests, especially in *Stegomyia*-free sites. Besides, mitigating CHIKV epidemics in suburban and rural areas intimately linked to the forested habitats is crucial to prevent virus establishment in the wild in the tropical Americas, if it is not already too late.
